# Prevention of Mother-to-Child Transmission and Early Real-Time DNA Polymerase Chain Reaction Results Among HIV-Exposed Infants in Bujumbura, Burundi

**DOI:** 10.24248/EAHRJ-D-18-00003

**Published:** 2018-11-23

**Authors:** Joseph Nyandwi, Sylvestre Bazikamwe, Désiré Nisubire, Pontien Ndabashinze, Mohamed Elsayed Shaker, Eman Said

**Affiliations:** a Hemodialysis Unit, Department of Internal Medicine, University Hospital of Kamenge, Bujumbura, Burundi; b Department of Gynecology and Obstetrics, University Hospital of Kamenge, Bujumbura, Burundi; c Microbiology Unit, Laboratory Department, University Hospital of Kamenge, Bujumbura, Burundi; d Department of Pediatrics, University Hospital of Kamenge, Bujumbura, Burundi; e Department of Pharmacology and Toxicology, Mansoura University, Mansoura, Egypt

## Abstract

**Background:**

Prevention of mother-to-child transmission (PMTCT) programmes aim to both eliminate vertical transmission of HIV and optimise the health and survival of infants born with HIV. Therefore, early infant diagnosis (EID) of HIV infection via DNA polymerase chain reaction (PCR) testing is a key component of PMTCT programming. We assessed the effectiveness of EID and PMTCT interventions at health-care facilities in Bujumbura, Burundi.

**Methods:**

This was a prospective analytical study of infants born to HIV-positive mothers on antiretroviral therapy (ART), who were followed from December 2016 to March 2017 at 3 centres providing PMTCT services in Bujumbura. Babies enrolled in this study received once-daily nevirapine from birth through to 6 weeks of life, after which HIV DNA PCR testing was conducted.

**Results:**

Of 122 HIV-exposed infants, 60 were boys and 62 were girls. The mother-to-child transmission rate at 6 weeks of life was 0.9%. Eighty-three (68%) of the women had commenced ART before pregnancy and 39 (32%) during pregnancy. The mean CD4 lymphocyte count was 653±308 cells/μl. Ninety-two (75.4%) of the pregnancies were planned, and 98 (80%) of the births were via spontaneous vaginal delivery. After birth, 111 (91.0%) infants were exclusively breastfed, and 11 (9.0%) infants received exclusive replacement feeding.

**Conclusion:**

There was a low rate of transmission of HIV from women taking ART to children who were given nevirapine for the first 6 weeks of life. Infants of HIV-positive women can live healthy lives free from HIV infection if their mothers participate in PMTCT programmes.

## INTRODUCTION

HIV/AIDS remains a disease of great public health importance, and vertical transmission of HIV – from mother to child – continues to be a common route of transmission, accounting for the vast majority of new infections in children.^1^ In 2012, about 330,000 children under 15 years of age worldwide were infected with HIV, according to estimates by the Joint United Nations Programme on HIV and AIDS (UNAIDS), with more than 90% of paediatric HIV infections occurring in sub-Saharan Africa.^1^ Most of these infections occurred during pregnancy, delivery, or breastfeeding, thereby making the prevention of mother-to-child transmission (PMTCT) an important public health strategy for reducing HIV transmission.^2^

PMTCT programmes provide antiretroviral therapy (ART) to HIV-positive pregnant women to prevent their infants from acquiring the virus. Effective PMTCT programmes require women and their infants to have access to and make use of a cascade of interventions, including antenatal services and HIV testing during pregnancy, ART for pregnant women living with HIV, safe childbirth and appropriate infant feeding practices, and infant HIV testing and other post-natal health-care services.^3^ In the absence of such interventions, the risk of mother-to-child transmission (MTCT) of HIV is 15% to 45%. However, ART and other effective PMTCT interventions can reduce this risk to below 5%.^4^

Early infant diagnosis (EID) permits the detection of HIV infection in exposed children from 4 to 6 weeks of age via a polymerase chain reaction (PCR) assay. In infants, HIV infection may progress to full-blown AIDS within the first few months of life. The advent of EID has brought about considerable survival benefits for HIV-infected infants who receive early ART.^5^ EID also permits the assessment of PMTCT programme effectiveness.

The Burundi PMTCT programme was implemented in 2000. The goal was to achieve virtual elimination of HIV infection in infants and young children. In 2011, the UNAIDS Global Plan was launched to reduce the number of new HIV infections acquired via MTCT by 90% by 2015.^6^ The World Health Organization (WHO) identified 22 priority countries, with the top 10 – including Burundi – accounting for 75% of the global PMTCT services need. It was estimated that the effective scale-up of interventions in these countries would prevent over 250,000 new infections annually. The 2010 Burundian Demographic and Health Survey reported a decrease of HIV prevalence in the general population from about 3% in 2007 to 1.4% in 2010 (1% in men and 1.7% in women).^7^ New HIV infections, acquired via MTCT, among children under 5 years of age accounted for 25% of all new infections during the same period. In 2016, Burundi had 84,000 people living with HIV, among whom were 4,300 pregnant women who received ART for PMTCT. WHO reporting estimated that fewer than 500 children were newly infected with HIV due to MTCT.^8^ Despite sustained PMTCT efforts, infants of HIV-positive women are at risk of becoming infected after birth. One of the major ways of reducing HIV transmission is by ensuring the consistent implementation of EID. The WHO recommends DNA real-time PCR (RT-PCR) HIV testing at 4 to 6 weeks of life for infants of HIV-positive mothers, and commencement of ART for HIV-positive children below 24 months of age.^1,2,9^

This study examined EID with DNA RT-PCR at 3 health facilities in Bujumbura, Burundi, with the aim of assessing the effectiveness of PMTCT interventions towards reducing vertical transmission of HIV.

## METHODS

### Study Design and Setting

This was a prospective analytical study of pregnant women enrolled in the Burundi PMTCT programme, implemented according to national guidelines. The study was conducted from December 2016 to March 2017 at 3 health facilities providing HIV screening, antenatal care, and PMTCT services in Bujumbura: the Buyenzi Community Medical Centre (CMC); the Society for Women Against AIDS in Africa (SWAA), Burundi; and the Burundian National Association of Support for People Living with HIV and AIDS Patients (ANSS).

The Buyenzi CMC is a public urban antenatal care clinic and was the first centre to implement PMTCT in Burundi in 2000.

The Burundi branch of the SWAA International was built in 1992 thanks to the initiative of a group of women wishing to fight the taboo and stigma attached to HIV/AIDS. It is a nonprofit organisation dedicated to HIV prevention and client support among women, children, families, and communities by providing medical and psychosocial support. At the end of June 2015, the SWAA sites in Burundi were monitoring 5,238 people, including 3,530 taking ART.

Founded in 1993, the Burundian ANSS was the first civil society organisation in Burundi to provide HIV prevention, care, and treatment services aimed at improving the well-being of people living with and affected by HIV. In 2016, 506 women were registered with the ANSS PMTCT programme.

### Study Population

This study focused on pregnant women living with HIV-1 infection and taking ART. Eligible women were those who were enrolled in PMTCT programmes at the 3 participating health-care facilities and expected to give birth within 6 weeks before the end of the study period. Babies delivered to enrolled women were included. Exclusion criteria among infants included being a second twin or a third triplet because of the reported lower MTCT risk among second twins and third triplets.^10^ CD4 cell counts were done during antenatal care. HIV-exposed infants received 2 mg/kg of once-daily nevirapine from birth for 6 weeks and were thereafter screened for HIV-1 by DNA RT-PCR.

### Blood Analysis

Blood analysis was carried out in the Department of Virology at the National Reference Laboratory of the National Institute of Public Health in Bujumbura. Whole blood samples (6 ml) drawn from the expectant mothers were collected in ethylenediaminetetraacetic acid (EDTA) tubes at the laboratories of each PMTCT service and directly transported to the National Institute of Public Health, where plasma was separated from the whole blood after centrifugation at 40,000 g for 10 minutes. The plasma was then stored in 1.5 ml aliquots at 80°C. CD4 T-cells were counted using the BD FACSCount System (Becton Dickinson, San Jose, CA, USA). At 6 weeks of life, all children born to enrolled mothers underwent qualitative DNA RT-PCR HIV testing. Dried blood spots from capillary samples were prepared on Whatman blotting papers and stored at room temperature until being used for DNA RT-PCR analysis with the Abbott RealTime HIV-1 Qualitative kit (Abbott Laboratories, Abbott Park, IL, USA). According to the manufacturer, the DNA RTPCR assay has a specificity of 100% and a sensitivity of at least 2,500 copies/ml using the dry blood spot procedure.

### Data Collection and Management

Data collection was conducted by a team of trained and supervised nurse data collectors. The principal investigator monitored data collection, and another senior member of the study team frequently conducted random checks to ensure data quality. Error and data consistency checks were conducted during analysis. Data were extracted from structured national data collection tools and entered into Microsoft Excel spreadsheets designed for data entry. The national tools from which the data for analysis were drawn included the PCR request and result forms as well as the EID register, containing information about the baseline characteristics of HIV-exposed babies, type of ART received by the mother, frequency of antenatal care follow-up, mode of delivery, mother's ART start date, infant feeding method, and outcome of the DNA RT-PCR test.

### Data Analysis

Data entered into the Microsoft Excel spreadsheet were cleaned and checked for consistency. The cleaned data were exported to Statistical Package for the Social Sciences (SPSS) for Windows, version 12.0 (SPSS Inc., Chicago, IL, USA), for data management and further statistical analysis. Frequency counts were performed to assess the completeness of all variables. The DNA RT-PCR result was the primary outcome variable and was determined for HIV-exposed infants at 6 weeks of life.

### Ethical Approval

This study was approved by the Comité d'Éthique et de Recherche en Santé Humaine (Committee of Ethics and Research in Human Health) of the Faculty of Medicine of the University of Burundi in accordance with the code of ethics for biomedical research involving human subjects (reference no. FM/CE/04/2016). Women were included in the study after they received a detailed explanation of the aim of the study and gave their written voluntary consent, when they were able to do so, and verbal consent if they were unable to read or write. The study used routinely collected, aggregated programme data at the 3 health-care facilities, and participant confidentiality was ensured, as personal identifiers – including names and identification numbers – were not recorded with the collected data.

## RESULTS

The study population was composed of 122 pregnant women and 122 babies. The mean age of the women was 31±6.6 years (range, 17 to 44 years); over half (n=62, 50.8%) of the women were aged between 30 and 39 years. Ninety-two (75.4%) of the pregnancies were planned, and 30 (24.6%) were unplanned. Eighty-three (68.0%) women commenced ART before pregnancy, and 39 (32.0%) began ART during pregnancy. The mean CD4 cell count for the women was 653±308 cells/μl, and 79 (64.7%) had ≥500 CD4 cells/μl. Only 1 (0.8%) mother gave birth at home, and the rest (n=121, 99.2%) delivered at health facilities (**Table 1**). Most (n=98, 80.3%) participants delivered via spontaneous vaginal delivery, with 24 (19.7%) delivering through caesarean section (**Table 2**).

**TABLE 1.** Baseline Characteristics of Women Enrolled in the Study (N=122)

Characteristics	n (%)
**Age (years)**	
<19	2 (1.6)
20–24	29 (23.8)
25–29	18 (14.8)
30–34	31 (25.4)
35–39	31 (25.4)
≥40	11 (9.0)
**Prepregnancy plan**	
Planned pregnancy	92 (75.4)
Unplanned pregnancy	30 (34.6)
**Place of delivery**	
Health-care facility	121 (99.1)
Home	1 (0.9)
**Timing of mother's antiretroviral therapy commencement**	
Before pregnancy	83 (68.0)
During pregnancy	39 (32.0)
**CD4 T-lymphocyte count (/μl) during antenatal care**	
≤200	2 (1.6)
201–349	17 (13.9)
350–499	24 (19.7)
≥500	79 (64.7)

**TABLE 2.** Selected Characteristics of HIV-Exposed Infants (N=122)

Characteristics	n (%)
**Sex**	
Male	60 (49.2)
Female	62 (50.8)
**Mode of delivery**	
Vaginal	94 (77.0)
Caesarean section	28 (23.0)
**Preterm delivery (<37 weeks of gestation)**	
No	122 (100)
Yes	0 (0)
**Feeding option adopted by mother**	
Exclusive breastfeeding	111 (91.0)
Exclusive replacement feeding	11 (9.0)
**HIV DNA RT-PCR result at 6 weeks of age**	
Positive	1 (0.8)
Negative	121 (99.2)

Abbreviation: RT-PCR, real-time polymerase chain reaction (assay).

All 122 babies were born at term (≥37 weeks of gestation); 60 (49.2%) of the newborns were boys, and 62 (50.8%) were girls. Most (n=111, 91.0%) of the infants received exclusive breastfeeding during the first 6 weeks of life; the other 11 (9.0%) children received exclusive replacement feeding. At 6 weeks of age, 121 (99.2%) infants tested negative for HIV by DNA RT-PCR, and 1 (0.8%) child tested positive (**Table 2**).

## DISCUSSION

Nearly all (n=121, 99.2%) of the enrolled infants tested negative by HIV DNA RT-PCR at 6 weeks of age. The 1 child who had a positive test was born at home and immediately transferred to the PMTCT centre, where nevirapine prophylaxis was initiated; however, the treatment did not continue at home. The MTCT rate of 0.8% is consistent with findings from a previous retrospective study conducted at Buyenzi CMC. In that study, Bindariye et al screened 774 children for PMTCT from January 2008 to December 2010, finding an MTCT rate of 1.4%.^11^ In another prospective follow-up study of 843 HIV-infected mothers from 2009 to 2011, the MTCT rate was 1.2%.^12^ This is also comparable to findings of 0.0% for Option B+ studies conducted in Burkina Faso and Ethiopia.^13,14^ Option B+ is a treatment approach that recommends immediate lifelong ART for all pregnant women living with HIV, regardless of CD4 count, and daily nevirapine or zidovudine for HIV-exposed infants from birth until 4 to 6 weeks of age, regardless of infant feeding method.^15^

Burundi, like several African countries, opted for Option B+ in 2014, which is why all expectant mothers enrolled in this study were already on the standard ART regimen, according to national guidelines.^15^ This may have contributed to the decline in the rate of HIV MTCT in Burundi. Eighty-three (68%) women were on lifelong ART before the pregnancy investigated in this study, and 39 (32%) women started treatment after testing positive for HIV during the applicable pregnancy. The mean CD4 count was 653±308 cells/μl, and only 2 (1.64%) women had CD4 counts <200 cells/μl. These levels could be due to antiretroviral drugs reducing maternal viral load and thereby reducing the risk of HIV transmission from mother to child by creating good conditions for the pregnancy. ART provides other antenatal and postnatal benefits; for example, in a Nigerian study, the rate of HIV transmission among infants of HIV-positive mothers was higher in babies whose mothers did not receive ART during pregnancy (28.6%) compared with those whose mothers commenced ART during pregnancy (5.4%), and it was lowest among those whose mothers commenced ART before pregnancy (3.4%).^16^ Our results are promising in the light of the further rollout of PMTCT interventions in sub-Saharan Africa, especially considering the most recent “test and treat”, or “treat all”, approach that recommends immediate initiation of treatment for all HIV-infected individuals, including pregnant women.^17^ However, high MTCT rates have still been reported relatively recently in Africa, specifically 6.3% at a tertiary hospital in Ado-Ekiti, Nigeria; 6.5% among public health-care facilities in Lusaka, Zambia; and 2.8% among public health-care facilities in South Africa.^16,18,19^

In our study, 98 (80.3%) women underwent vaginal deliveries and 24 (20%) underwent caesarean sections. This was in line with national guidelines that recommend against systematic caesarean deliveries for HIV-infected pregnant women. The frequency of caesarean section was largely determined by maternal and infant complications and not by maternal HIV status, and our caesarean section rate was similar to rates reported by other African studies.^16,20,21^

In our study, all babies were born at term. In a Nigerian study, the authors reported undesirable obstetric and neonatal outcomes in 48.3% of HIV-positive women compared to 30.3% of HIV-negative women. Preterm delivery and miscarriage were among the independent factors associated with HIV.^22^ In a Spanish study on the effect of highly active antiretroviral therapy (HAART) on spontaneous and iatrogenic preterm delivery, Lopez et al^23^ found that the incidence of prematurity was 19.7% in HIV-positive women and 8.5% in the control group. Prematurity secondary to treatment was significantly associated with the use of HAART during the second half of pregnancy. The incidence of spontaneous preterm birth was also higher among HIV-positive women not on HAART.^23^

WHO recommends antiretroviral prophylaxis for HIV-exposed neonates immediately after birth for 6 to 12 weeks.^9^ Nevirapine prophylaxis is recommended for 6 weeks for breastfeeding infants and for 4 to 6 weeks for infants who are not breastfeeding.^24^ In this study, all infants received nevirapine prophylaxis from birth to 6 weeks of life, regardless of whether they received exclusive breast-feeding (n=111, 91.0%) or exclusive replacement feeding (n=11, 9.0%). Exposed-infant antiretroviral prophylaxis serves as pre- and post-exposure prophylaxis and is especially protective against HIV acquisition via breastfeeding.^24–26^ A recent meta-analysis showed that HIV-exposed infants who do not receive antiretroviral prophylaxis at or after birth are more than 7 times more likely to become HIV-infected than infants who receive antiretroviral prophylaxis.^27^ Several studies have underscored the importance of infant antiretroviral prophylaxis in preventing MTCT of HIV.^24,25^

Despite all of these encouraging results, many studies have identified different maternal, obstetric, and child-level factors that determine antenatal, perinatal, and postnatal MTCT of HIV. Olana et al noted various predictive factors associated with positivity of DNA RT-PCR HIV testing at 6 to 8 weeks of age among children born to HIV-positive mothers: home delivery, maternal CD4 count <100 cells/ml during pregnancy, absence of PMTCT interventions for the mother, absence of prenatal consultations, and non-enrolment in an HIV-management programme during pregnancy.^14^ Mixed feeding, which has had reported rates of 5% in Botswana and Cameroon, has also been identified as an important risk factor for MTCT of HIV and infant deaths.^28,29^ Lack of participation in mother-to-mother support programmes, low partner involvement, poor ART adherence, positive syphilis test results, maternal malnutrition, and unplanned pregnancy have also been associated with MTCT of HIV.^30^

### Limitations

Our study's limitations include the short study period. A longer study period could have allowed for the inclusion of more participants, leading to more consistent results. The study was carried out at 3 centres with broad experience in managing PMTCT interventions. The centres are located in Bujumbura, where accessibility to heath facilities is high compared with the rest of the country. Our results, therefore, are not generalisable to the entire country. Additionally, this study focused only on EID of HIV at 6 weeks of age. Future studies should evaluate the effectiveness of PMTCT programmes until HIV-exposed infants reach 18 months of age.

## CONCLUSION

This study highlighted the low rate of MTCT of HIV when HIV-positive pregnant women receive ART and the infants born to these women receive nevirapine for the first 6 weeks of life. Infants born to HIV-positive women can live healthy lives free from HIV infection if their mothers participate in PMTCT programmes. To identify infants with HIV and monitor those at risk of infection, HIV testing and counselling programmes focused on PMTCT interventions should be scaled up in antenatal, labour and delivery, and postnatal settings. Also, institutional and community-based comprehensive health education programmes that emphasise the importance of skilled birth attendance, postpartum care, and PMTCT interventions are essential. This calls for the Ministry of Public Health and other concerned partners to work towards improving the PMTCT programme by making DNA RT-PCR testing available and accessible to all families of exposed infants for early HIV diagnosis.
